# Parental income and mental disorders in children and adolescents: prospective register-based study

**DOI:** 10.1093/ije/dyab066

**Published:** 2021-05-11

**Authors:** Jonas Minet Kinge, Simon Øverland, Martin Flatø, Joseph Dieleman, Ole Røgeberg, Maria Christine Magnus, Miriam Evensen, Martin Tesli, Anders Skrondal, Camilla Stoltenberg, Stein Emil Vollset, Siri Håberg, Fartein Ask Torvik

**Affiliations:** 1 Norwegian Institute of Public Health, Oslo, Norway; 2 University of Oslo, Oslo, Norway; 3 University of Bergen, Bergen, Norway; 4 Institute for Health Metrics and Evaluation, University of Washington, Seattle, USA; 5 Frisch Centre, Oslo, Norway; 6 MRC Integrative Epidemiology Unit at the University of Bristol, Bristol, UK; 7 Population Health Sciences, Bristol Medical School, Bristol, UK; 8 NORMENT, Oslo University Hospital, Oslo, Norway; 9 GSE, University of California, Berkeley, CA, USA

**Keywords:** Mental disorders, income, inequality, childhood, adolescence

## Abstract

**Background:**

Children with low-income parents have a higher risk of mental disorders, although it is unclear whether other parental characteristics or genetic confounding explain these associations and whether it is true for all mental disorders.

**Methods:**

In this registry-based study of all children in Norway (*n* = 1 354 393) aged 5–17 years from 2008 to 2016, we examined whether parental income was associated with childhood diagnoses of mental disorders identified through national registries from primary healthcare, hospitalizations and specialist outpatient services.

**Results:**

There were substantial differences in mental disorders by parental income, except for eating disorders in girls. In the bottom 1% of parental income, 16.9% [95% confidence interval (CI): 15.6, 18.3] of boys had a mental disorder compared with 4.1% (95% CI: 3.3, 4.8) in the top 1%. Among girls, there were 14.2% (95% CI: 12.9, 15.5) in the lowest, compared with 3.2% (95% CI: 2.5, 3.9) in the highest parental-income percentile. Differences were mainly attributable to attention-deficit hyperactivity disorder in boys and anxiety and depression in girls. There were more mental disorders in children whose parents had mental disorders or low education, or lived in separate households. Still, parental income remained associated with children’s mental disorders after accounting for parents’ mental disorders and other factors, and associations were also present among adopted children.

**Conclusions:**

Mental disorders were 3- to 4-fold more prevalent in children with parents in the lowest compared with the highest income percentiles. Parents’ own mental disorders, other socio-demographic factors and genetic confounding did not fully explain these associations.

**Key Messages**

- Mental disorders in children decreased continuously with increasing parental income for all mental disorders, except eating disorders.
- The parental-income gradient was largest for attention-deficit hyperactivity disorder, followed by anxiety and depression.
- Our study suggests that associations between lower parental income and children’s mental disorders were partly, but not fully, attributed to other socio-demographic factors, parents’ own mental disorders and genetic factors.

## Introduction

Children from low-income households are at greater risk for poor health outcomes, including childhood mental disorders.^1–5^ Nevertheless, we have an inadequate understanding of the pathways between parental income and mental disorders, and there is a need to better elucidate the mechanisms.^6^

Survey data are commonly used to determine patterns of mental disorders in relation to income, although the prevalence of mental disorders and parental income are not well measured in surveys.^7^^,^^8^ Reporting and response biases affect the accuracy of both variables, leading to questionable validity and difficulties with comparisons between studies.^7–10^ The use of national registry data in studies of parental income and offspring mental health has been limited, and can produce biased results, as access to mental healthcare is highly income-dependent in many countries.^11^^,^^12^

Because of these challenges, there are several remaining questions. First, it is not known whether the prevalence of children’s mental disorders is exclusively decreasing with income, as it has been suggested that some mental disorders may increase for the highest incomes.^13^ Second, it is not known whether the association with parental income differs according to the type of mental disorder. Third, other factors such as region of residence, socio-demographic characteristics and genetic predispositions are likely to influence associations, but the relative importance of these factors remains largely uncharacterized.^14^^,^^15^

In Norway, mental healthcare is free for all children aged ≤18 years.^16^ Hospital admissions and inpatient treatment are also free, whereas primary-care visits are free for all children aged ≤16 years and thereafter are mostly subsidized. Thus, bias from income-dependent access to healthcare is small. National registries include information on all primary healthcare contacts, hospitalizations and specialist outpatient services for mental disorders and can be linked to tax records to obtain independent information on parental-income levels.

Children’s diagnoses linked with tax records of parents were used to characterize the association between parental income and childhood mental disorders by sex and age, and to investigate how associations between parental income and mental disorders in children were influenced by other determinants, such as parental mental disorders, socio-demographic factors or other heritable factors.

## Methods

### Data sources and study population

The study population included all children aged 5–17 years from 2008 to 2016 in Norway. Linked individual-level information was retrieved from five Norwegian national registries. These data provided information on parental income and educational level, as well as mental-disorder diagnoses from primary and specialist healthcare. Data from diagnostic interviews and on healthcare use, linked with income from tax records, were obtained from the Survey of Health and Living Conditions (SHLC)^17^ (Part III in the Supplementary Material, available as Supplementary data at *IJE* online).

Children with parents in the lowest 2% income group were removed, as these parents with very low or negative income may have income from unregistered sources or expenses covered by other means.^18^ Persons with immigrant backgrounds have lower rates of mental healthcare use than non-immigrants, despite an equal or greater need,^19–21^ and hence children born outside Norway or with parents born outside Norway were not included in the primary study population, but studied separately (see Part V in the Supplementary Material, available as Supplementary data at *IJE* online).

The data also allowed us to construct a subgroup of adopted children born in South Korea, China or Columbia.^22^ Refer to Part II in the Supplementary Material, available as Supplementary data at *IJE* online, for details on how adopted children were defined.

### Income

Income data were obtained from tax records and included income from wages, self-employment, capital income, pensions and government assistance such as disability benefits. Parental income was determined as the sum of both parents’ income after taxes and adjusted for inflation using the Norwegian consumer price index.^23^ For each calendar year, children in the study population were assigned to a percentile ranked from 1 to 100 based on total parental income relative to that of all other children of the same sex and age. In some analyses, income levels were grouped into quartiles or deciles to facilitate comparisons.

### Diagnoses of mental disorders

Diagnoses of mental disorders were obtained from reimbursement data from primary healthcare, hospitalizations and specialist outpatient services in the Norwegian Control and Distribution of Health Reimbursement (KUHR) database and the National Patient Registry (NPR).^24^^,^^25^ These include data from primary-care physicians, psychologists, specialist psychologists, emergency rooms and contracting specialist physicians; all hospitals; mental healthcare facilities for adults; mental healthcare facilities for children and youth; injuries treated in hospital and municipal emergency departments; specialized interdisciplinary addiction treatment; and private rehabilitation institutions.^26^

Diagnostic data for ≤21 health conditions were extracted from specialist care for the children and their parents. In primary care, two (primary and secondary) diagnoses for each case were also extracted. Mental disorders are registered in specialist care with the International Classification of Diseases, Tenth Revision (ICD-10), whereas the International Classification of Primary Care version 2 (ICPC-2) coding system (Part VI in the Supplementary Material, available as Supplementary data at *IJE* online) is used in primary care.^27^^,^^28^ By excluding symptoms and non-disease codes from ICPC-2 prior to translating the ICPC-2 codes into ICD-10 (Table 1), only diagnosed mental disorders were included in this study.^29^ Parental mental disorders (dummy) indicated whether any of the parents had any of the mental disorders in listed in Table 1.

**Table 1:** Categorizations of mental disorders according to the ICD-10 and ICPC-2

Mental disorder	ICD-10 code(s)	ICPC-2 code(s)	1-year period prevalent cases per 100 000 for 2016 (95% CI)	9-year period prevalent cases per 100 000 for 2008–2016 (95% CI)
Substance use	F10–19		61.2 (54.9, 67.5)	235.02 (217.04, 253)
Psychotic disorders (including schizophrenia)	F20–29	P72, P98	42.43 (37.19, 47.68)	163.62 (148.61, 178.62)
Bipolar disorder	F30-F31	P73	32.29 (27.71, 36.87)	102.26 (90.4, 114.13)
Depressive disorders	F32-F33	P76	908.18 (884.01, 932.36)	3189.49 (3124.25, 3254.72)
Anxiety disorders	F40–44, F93–93.2	P74, P79, P82	1616.2 (1584.07, 1648.34)	5740.28 (5653.92, 5826.64)
Somatoform disorders	F45	P75	82.5 (75.18, 89.82)	538.58 (511.4, 565.75)
Eating disorders	F50	P86	154.01 (144.02, 164.01)	510.23 (483.78, 536.68)
Idiopathic developmental intellectual disability	F70–79	P85	341.84 (326.96, 356.71)	787.23 (754.42, 820.05)
Autism-spectrum disorders	F84		499.06 (481.1, 517.02)	1357.03 (1314.07, 1399.98)
Attention-deficit hyperactivity disorder (ADHD)	F90	P81	2929.96 (2886.98, 2972.94)	6426.33 (6335.29, 6517.38)
Conduct disorder	F91–92		229.58 (217.39, 241.78)	1108.73 (1069.86, 1147.61)
Mental disorders with typical childhood onset (including social function and tic disorders, but excluding ADHD)	F94, F95, F98		1096.35 (1069.81, 1122.88)	4478.34 (4401.55, 4555.13)
Code F99 not specified mental disorder	F99	P99, P77	664.74 (644.03, 685.45)	2624.36 (2565.01, 2683.71)
Other mental disorders	F03–09, F34, F38, F39, F45–49, F51–52, F55–55.8, F56–69, F80–83, F85–89	P70, P71, P78, P80	472.01 (454.55, 489.48)	2334.43 (2278.38, 2390.49)

### Other socio-demographic variables

The geographical indicator variable (394 regions based on city districts and municipalities) was taken from the Population Register.^30^ The number of household members (indicator variables), mother and father employment (dummy) and single-parent household (dummy) were based on income and household data from the National Registry for Personal Taxpayers. Parental education (indicator variables for basic/secondary/tertiary) was from the National Education Database.^31^

### Statistical methods

To characterize the association between parental income and the prevalence of children’s mental disorders, the pooled prevalence of mental disorders in children was calculated for each parental-income percentile [with 95% confidence intervals (CIs)] based on pooled data with yearly observations of mental disorders and parental-income rank.^32^ To estimate the unadjusted associations of parental income with 14 subgroups of mental disorders in children, the odds ratios (ORs) for each category of mental disorders (dichotomized) were estimated by logistic regression with a continuous variable for parental income.^32^ Further details on all statistical methods are described in Part I of the Supplementary Material, available as Supplementary data at *IJE* online.

To evaluate determinants of the association of mental disorders with parental income, ORs adjusted for parental characteristics were estimated by logistic regression for any mental disorder (dichotomized) on continuous parental-income percentiles and reported for every decile increase in parental income. Adjustments were made for geographical indicator variables, mother’s age, father’s age, number of household members, mother and father employment, single-parent household, parental education and parental mental disorders. These adjustments were done separately as well as jointly to indicate the impact of each factor on the differences in offspring mental disorders according to parental income (Part I in the Supplementary Material, available as Supplementary data at *IJE* online).

To evaluate whether mental disorders are related to area income inequality, adjusted ORs were estimated by regressing the 1-year prevalences of mental disorders on the Gini coefficient, P90/P10, and on the proportion below the Organisation for Economic Co-operation and Development (OECD) 60 poverty line, within 133 regions of residence defined by city districts and municipalities in Norway, using multilevel mixed-effects logistic regression and area-level fixed-effects logit-regression models.

In sub-analyses, associations of mental disorders and parental income were estimated in international adoptees who are genetically unrelated to their parents using logistic regression adjusted for age, sex and birth year interacted with country of birth (Part II in the Supplementary Material, available as Supplementary data at *IJE* online).^32^

All analyses were done on individual-level data and cluster robust standard errors were used to account for multiple observations of the same individual across years in all models (Part I in the Supplementary Material, available as Supplementary data at *IJE* online).^32^

Psychological distress, from diagnostic interviews, was regressed against health-service use and interacted with parental income, using logistic regression (see Part III in the Supplementary Material, available as Supplementary data at *IJE* online).

## Results

In total, 1 354 393 children, aged 5–17 years, comprising 7 261 964 person-years from 2008 to 2016 were included in the study. After excluding 293 647 children with immigrant parents and 5698 internationally adopted children, the primary study population consisted of 969 206 children, comprising 5 199 742 person-years (Supplementary Table S1, available as Supplementary data at *IJE* online). The mean age was 11.2 years (SD = 3.7). Information on parental income was available for >99.9% of the study population. The median parental income after tax was USD 69 489.

### Association of parental income and mental disorders by sex and age

**Figure 1:**
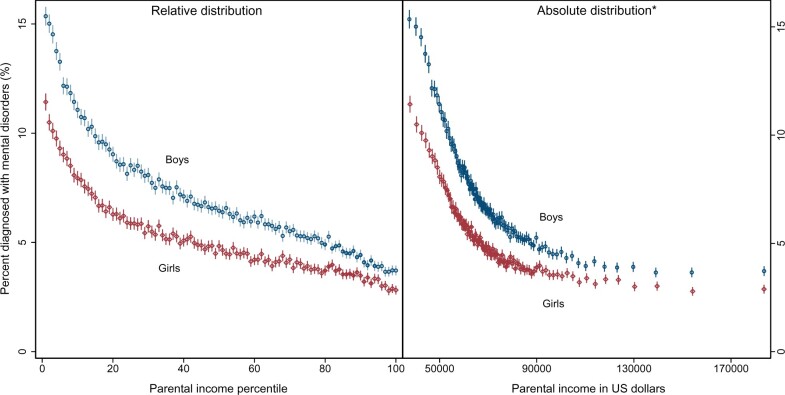
Proportion of children aged 5–17 years with any mental disorders by parental income and sex, 2008–2016. Estimations were based on all residents aged 5–17 years in Norway for 2008–2016, excluding individuals with parents with the lowest 2% income and individuals with immigrant backgrounds (969 206 children). Bars represent 95% confidence intervals on point estimates, using robust standard errors clustered by individuals. *The top income percentile was omitted for scaling purposes. The mean parental income in the top income percentile was US$ 395 594 and 3.7% of boys and 2.8% of girls were diagnosed with mental disorders. Norwegian kroner were translated into US dollars using the 2011 individual consumption expenditure by household value of 9.797 from the International Comparison Program (http://www.worldbank.org/en/programs/icp#5). Bars represent 95% confidence intervals on point estimates, using robust standard errors clustered by individuals. The solid lines represent the predicted percentage of mental disorders from restricted cubic splines with seven knots of parental-income percentiles.

Higher parental income was associated with lower prevalence of all mental disorders in both sexes (Figure 1, left). Proportions with mental disorders decreased steeply between the 1st and 20th percentiles of parental income, with a steady but more modest decrease from the 21st to the 99th percentiles. The statistical relationships for prevalence of all mental disorders with *absolute* income decreased steeply from the lowest incomes up to ∼70 000 USD and then flattened (Figure 1, right). The variations in children’s mental disorders were larger by paternal rather than maternal income levels, as shown in Supplementary Figure S1, available as Supplementary data at *IJE* online.

**Figure 2:**
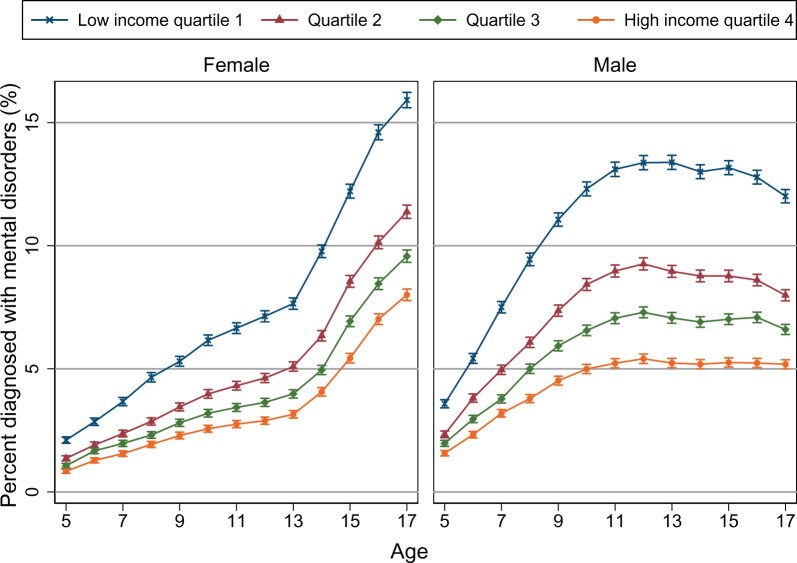
Any mental disorders by age, sex and parental-income quartile. Estimations were based on all residents aged 5–17 years in Norway for 2008–2016, excluding individuals with parents with the lowest 2% income and individuals with immigrant backgrounds (969 206 children). Bars represent 95% confidence intervals on point estimates, using robust standard errors clustered by individuals. The solid lines represent the predicted percentage of mental disorders from restricted cubic splines with seven knots of parental-income percentiles.

Prevalence by age differed between boys and girls (Figure 2). Prevalence of all mental disorders in girls was higher with increasing age in all income groups, with a steeper slope seen for ages 13–17 years compared with 5–12 years. In boys, the greatest rate of increase for all mental disorders was between 5 and 12 years. For girls and boys, at each age assessed, the prevalence of all mental disorders was higher in children with lower parental income. The largest gap in prevalence by parental income was at age 17 years for girls and at age 12 years for boys.

### Association of parental income and subcategories of mental disorders

**Figure 3:**
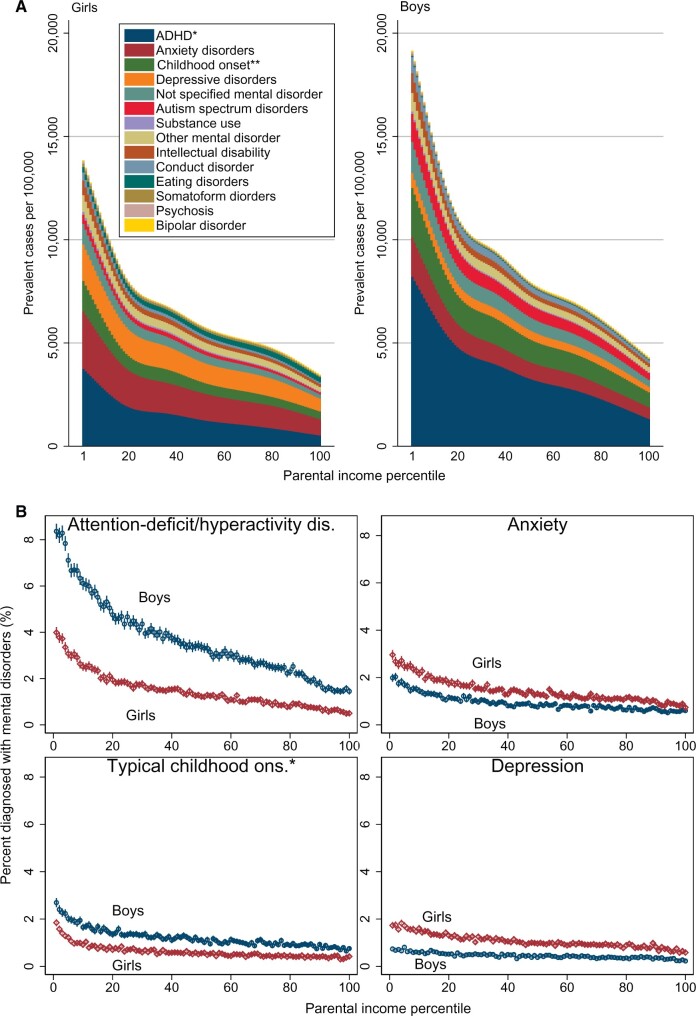
Proportion of children aged 5–17 years with mental disorder by parental income and disorder subgroup, 2008–2016. (A) Mental disorders by parental-income percentile, sex and disorder subgroup. (B) Subgroups of mental disorders by child sex. *Attention-deficit hyperactivity disorder. **Mental disorders with typical childhood onset (including social function and tic disorders, but excluding ADHD). Estimations were based on all residents aged 5–17 years in Norway for 2008–2016, excluding individuals with parents with the lowest 2% income and individuals with immigrant backgrounds (969 206 children). Bars represent 95% confidence intervals on point estimates, using robust standard errors clustered by individuals.

**Figure 4:**
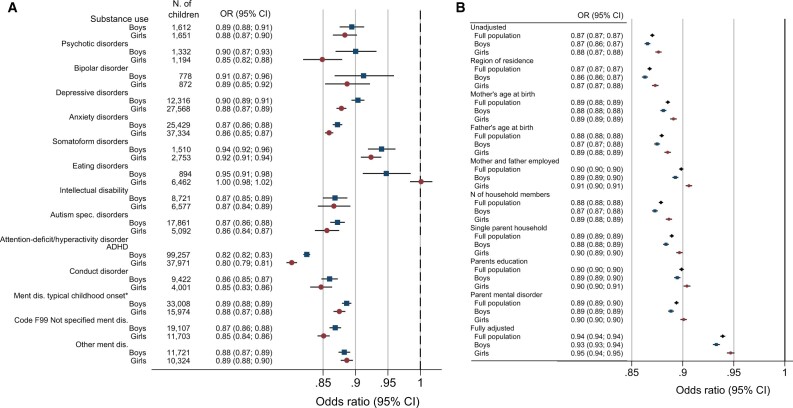
Odds ratios for childhood mental disorders for every 10% higher level of parental income. (A) Associations between children’s diagnosed mental disorders and parental-income percentile. *Mental disorders with typical onset in childhood from ICD10 codes F90–99, including social function and tic disorders, but excluding ADHD. Estimations were based on all residents aged 5–17 years in Norway for 2008–2016, excluding individuals with parents with the lowest 2% income and individuals with immigrant backgrounds (969 206 children). (B) Odds ratios for any mental disorders for children and parental-income deciles adjusted for eight covariates. Estimations were based on all residents aged 5–17 years in Norway for 2008–2016, excluding individuals with parents with the lowest 2% income and individuals with immigrant backgrounds (969 206 children). Bars represent 95% confidence intervals on point estimates, using robust standard errors clustered by individuals. Separate adjustments were initially done for 394 geographical identifiers based on municipalities and city districts, mother’s age (1-year age groups), father’s age (1-year age groups), number of household members (categorical), mother and father employed (yes/no), single-parent household (yes/no), parental education (basic/secondary/tertiary) and parental mental disorders (yes/no), before simultaneous adjustment for all covariates was undertaken (Part I in the Supplementary Material, available as Supplementary data at *IJE* online).

Attention-deficit hyperactivity disorder (ADHD) was the largest contributor to the absolute difference in mental disorders by parental income in boys, whereas anxiety and depression were the main contributors in girls (Figure 3A and B). With each decile increase in income, the ORs were lower for all subcategories of mental disorders, except for eating disorders in girls (Figure 4A and Supplementary Figure S4, available as Supplementary data at *IJE* online). The ORs were highest for ADHD in both boys and girls (Figure 4A).

### Evaluation of factors associated with differences in mental disorders by parental income

**Figure 5:**
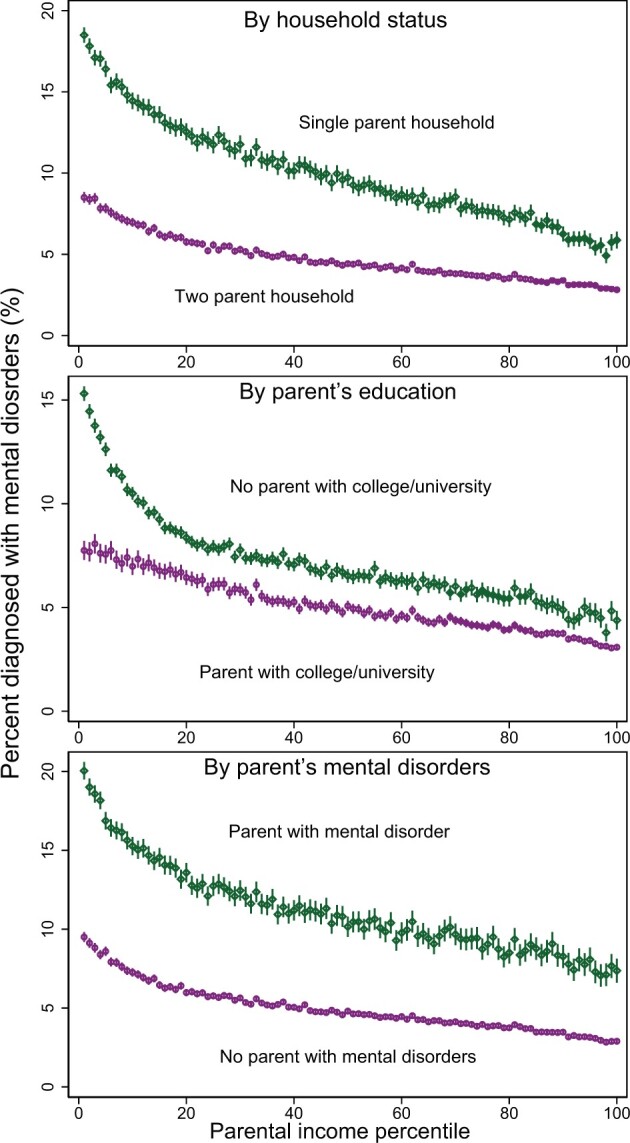
Proportion of children aged 5–17 years with any mental disorders by parental income and parental characteristics, 2008–2016

The prevalence of any mental disorders was higher among children in one-parent households than among children in two-parent households (Figure 5). This difference was larger at lower income percentiles.

There was a higher prevalence of any mental disorders among children whose parents had mental disorders compared with children of parents with no diagnosed mental disorder (Figure 5). This difference was larger at lower income levels. There was also a higher prevalence of any mental disorders in children whose parents had low levels of education compared with those with higher education at all income levels. This difference was also larger at lower income levels (Figure 5). Supplementary analyses suggested that parental income explained slightly more of the variance in children’s mental disorders than did parental education (see more details in Part IV in the Supplementary Material, available as Supplementary data at *IJE* online).

Compared with the ORs from univariate regressions, the decreases in ORs of mental disorders with deciles of parental income were not influenced by adjustment for region of residence, but some attenuation was observed when adjusting for the mother’s and father’s age, number of household members and single-parent households. The greatest attenuation was observed for adjustments for parental employment status and education (Figure 4B).

**Figure 6:**
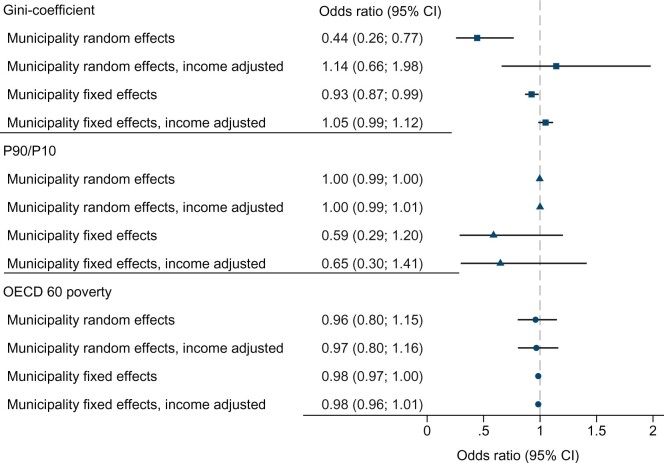
Associations between children’s diagnosed mental disorders and local area income inequality and poverty. Adjusted ORs for area-level characteristics were estimated by regressing the 1-year prevalence of mental disorders on two measures of income inequality—the Gini coefficient and P90/P10—and the proportion below the Organisation for Economic Co-operation and Development (OECD) 60 poverty line, within 133 regions of residence, using multilevel mixed-effects logistic regression and area-level fixed-effects logit-regression models. Two versions of each model were estimated: one model adjusted for calendar year, age, age squared, sex and interactions between age and sex; and one model that also adjusted for parental income and aggregate area income.

There was no difference in the prevalence of childhood and adolescent mental disorders according to the income inequality in their region of residence (Figure 6).

**Figure 7:**
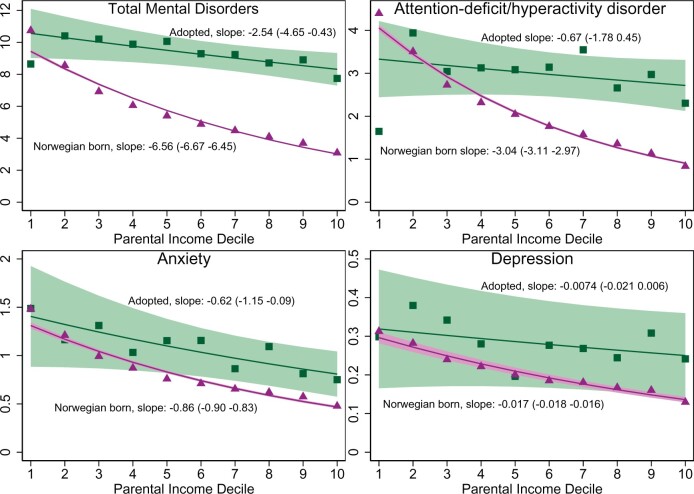
Mental disorders by income deciles in international adoptees and Norwegian-born children, 2008–2016. Estimations were based on two populations: (i) all residents aged 5–17 years in Norway for 2008–2016, excluding individuals with parents with the lowest 2% income and individuals with immigrant backgrounds (969 206 children); and (ii) international adoptees from China, South Korea and Columbia aged 5–17 years in Norway for 2008–2016, excluding individuals with the lowest 2% income (5698 children). The 1-year prevalence of mental disorders with 95% confidence intervals for each parental-income percentile were estimated separately within populations (i) and (ii). These were estimated using pooled data across all years, by generalized estimating equations with a logit link function and an independent working-correlation structure. The regressions were adjusted for age, sex and birth year interacted with country of birth (Part II, Equations (4) and (5) in the Supplementary Material, available as Supplementary data at *IJE* online). Robust standard errors were used to account for multiple observations of the same individual across years. Shaded areas are 95% confidence intervals using these standard errors.

Higher parental income was associated with lower prevalence of children’s mental disorders, also in the international adoptee subgroup, although with a less pronounced association, compared with the Norwegian-born. For every decile increase in parental income, there were 0.25% (95% CI: −4.7, 0.4) fewer adoptees diagnosed with mental disorders compared with 0.66% fewer (95% CI: −0.67, 0.65) per decile in Norwegian-born children. Except for eating disorders, international adoptees had ∼1.5–2 times higher prevalence of any mental disorders compared with Norwegian-born children (Figure 7 and Supplementary Table S1, available as Supplementary data at *IJE* online).

### Income and access to care

Psychological distress, as measured in diagnostic interviews, in children was significantly associated with health-service use, but there was no difference in the strength of this association over parental income (Part III in the Supplementary Material, available as Supplementary data at *IJE* online).

## Discussion

Three major conclusions can be drawn from this study. First, despite relatively equal access to health services, childhood mental disorders were found to decrease continuously with parental income and there was no dividing line above or below which additional income was no longer associated with mental disorders. The associations varied with child age and sex. Second, the association with parental income was present for all mental disorders except eating disorders and largest for ADHD. Third, the association of parental income with mental disorders could partly, but not fully, be attributed to parental mental disorder and socio-demographic factors. In addition, the associations were present, but less pronounced, in children genetically unrelated to their parents.

### Association of parental income and mental disorders by sex and age

The observed patterns of association and sex differences are similar to those of differential life expectancy by income in adults aged ≥40 years in Norway.^18^ This supports the suggested link between childhood family income and the subsequent socio-economic inequalities in health in adults.^33^

### Association of parental income and subcategories of mental disorders

Previous studies have found associations between parental income and selected mental disorders in children.^1^ However, studies covering a range of categories are lacking. This study found that the most pronounced associations with parental income were for ADHD in both boys and girls. The prevalence of eating disorders did not vary with parental income in girls. Although varying associations were detected, these findings may be related to the pervasive co-morbidity within mental disorders.^34^

### Evaluation of factors associated with differences in mental disorders by parental income

This study replicates previous findings that one-parent households, low parental education and mental disorders in parents are factors associated with children’s mental disorders.^1^^,^^35^^,^^36^ Further, the results show that absolute differences in mental disorders by single-parent household status, parental education and parental mental disorders were greater in children with parents at lower income levels.

Associations between parental income and children’s mental disorders were attenuated when adjusted for household and parental characteristics such as age, education, employment status, mental disorders and one-parent household. Nonetheless, adjusted parental income remained an independent predictor for mental disorders in children, which is in line with previous findings.^3^

The influence of a genetic component is also suggested. Children of parents with mental illness are at a higher genetic and environmental risk of developing psychopathology.^37^^,^^38^ Low income can be a consequence of psychopathology in parents.^37^ The largest income difference was found for ADHD, a mental disorder with a strong heritable component, which is also associated with reduced income in adulthood.^38^ In contrast, the difference across the income spectrum was smaller for anxiety, which has been shown to have a large environmental component.^38^ These differences suggest confounding by underlying genetic susceptibility on the relationship between parental income and offspring mental disorders. In addition, the associations between parental income and mental disorders in adopted children were weaker compared with children living with their biological parents. The differences in the associations with parental income observed among adopted children and Norwegian-born children were also greater for ADHD than for anxiety disorders.

Although weaker than in children living with their biological parents, the statistically significant associations between parental income and mental disorders in adopted children support that at least some mental health problems are a result of social factors.^3^

Studies from other countries suggest that registries do not fully capture interview-based diagnoses in children from lower-income families.^11^ If parental income is associated with use of health services for mental disorders given equal need, diagnoses from health registries could be biased indicators of income gradients in mental disorders. To explore this, we conducted supplementary analyses of the association between psychological-distress score, from the SHLC Survey,^17^ and health service. This analysis did not suggest that this bias the estimates for Norway.

Also, a strength of our study was that we used primary-care data in addition to specialist-care data, whilst most prior studies have included only specialist services.^5^ Furthermore, comparisons of diagnostic data from the Composite International Diagnostic Interview with health registry diagnoses on major depressive and anxiety disorders in Norway have been published previously.^8^ As indicators, registry-based diagnoses have moderate sensitivity and excellent specificity, with 0.2–4.2% false positives.^8^ The health survey and registry data used in this study have been found to measure the same symptoms.^8^

This study has some limitations. First, as the diagnoses of mental disorders in children were obtained from health registries, information was only available for individuals in contact with health services. Individuals with less severe cases of depressive disorders and anxiety do not all seek care.^8^^,^^39^ Thus, children with mild or transient symptoms may be underrepresented. Second, primary and specialist healthcare use different standards of diagnostic codes. ICPC2, used in primary care, relies on broader diagnostic categories than the ICD-10 used in specialist care. Thus, some specific mental disorders, such as those in the autism spectrum, do not have specific codes in the primary-care database. In Norway, however, children with autism and other severe conditions are unlikely to not have been under specialist care during the study period. Third, particularities of the setting and potential non-random assignment of adopted children to adoptive parents can affect the interpretation of data on the association between income and mental disorders in adopted children (Part II in the Supplementary Material, available as Supplementary data at *IJE* online).

## Conclusions

In Norway, a country with universal public healthcare, there were substantial differences in mental disorders in children by parental income. Income-related differences in children’s mental disorders were partially attributable to parents’ own mental disorders and socio-demographic characteristics. The results therefore represent differences that might be higher in countries with weaker health and welfare systems for those at lower income levels.

## Supplementary data

Supplementary data are available at *IJE* online.

## Ethics approval

This study was approved and participant consent was waived by the Regional Committee for Medical and Health Research Ethics South-East Norway, reference number 2013/2394.

## Funding

This work was funded by the Research Council of Norway [project numbers 262030, 300668 and 262700] and by the Norwegian Institute of Public Health. The funders had no role in the design and conduct of the study; collection, management, analysis and interpretation of the data; preparation, review or approval of the manuscript; and decision to submit the manuscript for publication.

## Data availability

The data underlying this article were provided by Statistics Norway, the Norwegian Directorate of Health and the Norwegian Institute for Public Health by permission. Researchers can gain access to the data by submitting a written application to the data owners at www.helsedata.no and https://www.ssb.no/en/omssb/tjenester-og-verktoy/data-til-forskning.

## Supplementary Material

dyab066_Supplementary_Data
