# Supplementary material for: Parental income and mental disorders in children and adolescents: prospective register-based study
Source: Int J Epidemiol. 2021 May 11;50(5):1615–27. doi: 10.1093/ije/dyab066 (PMC8580274; doi:10.1093/ije/dyab066)
Supplement: dyab066_Supplementary_Data [file dyab066_supplementary_data.docx]

**Supplementary Appendix**

Parental income and mental disorders in children and adolescents in Norway: prospective register-based study

This supplementary material provides further details on methods and additional analyses related to the main paper.

**Supplemental Methods**

**Part I:** Statistical methods

**Part II:** Associations of adoptive parental income and children’s mental disorders in international

adoptees

**Part III:** Variation in the association between mental distress and health service use by parental income

in children and adolescents

**Part IV:** The proportion of variance in the child-adolescent mental disorders predictable by income and

education

**Part V:** Comparison of Diagnosed Mental Disorder by Immigrant Categories

**Part VI:** International Classification of Primary Care, Second edition (ICPC-2)

**Part VII:** Associations between Children’s Diagnosed Mental Disorders and Local Area Income

Inequality and Poverty

**Supplemental Results**

**Table S1**. Sample characteristics

**Table S2.** Adjusted Odds Ratios for Health Service Use for Participants by Mental Distress Score,

Parental Income and Interactions between Mental Distress Scores and Parental Income^a^

**Table S3**. Adjusted Odds Ratios for Health Service Use for Participants by Mental Distress Dummy,

Parental Income and Interactions between Mental Distress Dummy and Parental Income^a^

**Table S4.** Percent of the main estimation sample within each education and income group

**Table S5.** Mental disorders by immigrant background

**Figure S1.** Prevalence of Child-Adolescent Mental Disorders and mothers and fathers income

**Figure S2.** Prevalence of Child-Adolescent Mental Disorders and parental income percentile in the

population with immigrant background

**Figure S3.** Prevalence of Child-Adolescent Mental Disorders by parental income percentile in the main

estimation sample for 14 different categories of disorders.

**Figure S4.** Prevalence of Child-Adolescent Mental Disorders by Parental Education

**Figure S5**. Prevalence of Child-Adolescent Mental Disorders by seven Parental Income Groups

Authors:

Jonas Minet Kinge, Simon Øverland, Martin Flatø, Joseph Dieleman, Ole Røgeberg, Maria Christine Magnus, Miriam Evensen, Martin Tesli, Anders Skrondal, Camilla Stoltenberg, Stein Emil Vollset, Siri Håberg, Fartein Ask Torvik

**Part I: Statistical methods**

This part provides a more detailed description of the statistical methods.

Poled prevalence of mental disorders with 95% confidence intervals for each parental income percentile were estimated separately within subpopulations defined by sex, household status, education, and parents own mental disorders. The prevalences were estimated by regressing dichotomous variables for yearly observations of mental disorders on dummy variables for time-varying parental income percentiles, using pooled data across all years. The estimation used generalized estimating equations (GEE) with a logit link function and an independent working correlation structure. Robust standard errors were used to account for multiple observations of the same individual across years, by the approach from Liang & Zeger.^1 2^ The model can be executed by the Stata command:

xtgee y x, family(binomial) link(logit) corr(independent)

Formally, the model can be written as follows:

$logit\{Pr \left( {MentalDisorder}_{it}=1 | x_{it} \right)\}=\alpha+\beta_{1}{IncCentile2}_{it}+\ldots+\beta_{99}{IncCentile100}_{it}$, (1)

${MentalDisorder}_{it}$is a dummy variable that is equal to 1 when the child had any mental disorder in year (t) and zero otherwise; and, income percentiles were included as 99 dummy variables, omitting the first income percentile.

Adjusted odds ratios (ORs) for mental disorders by parental income were estimated by regressing dichotomous variables for mental disorders on continuous parental income percentile. Separate adjustments were completed for 394 geographical identifiers, mother’s age (one year age groups), father’s age (one year age groups), number of members in child’s household (categorical), mother’s and father’s employment (yes/no), the child’s household being a single parent household (yes/no), highest education level obtained by a parent (basic/secondary/tertiary), and mental disorder in one or both parents (yes/no). These adjustments were completed separately as well as jointly to indicate the impact of each association between income and mental disorders. The model can be written as:

$logit\{Pr \left( {MentalDisorder}_{it}=1 | x_{it} \right)\}=\alpha+\beta_{1}{IncPercentile}_{it}+X_{it}^{'}\gamma$, (2)

Where parental income percentile is included as a continuous variable and $X_{it}^{'}$ is a vector of control variables. Robust standard errors were used to account for multiple observations of the same individual across years, by the approach from Liang & Zeger.^1 2^

**Part II: Associations of adoptive parental income and children’s mental disorders in international adoptees**

Prevalence of mental disorders with 95% confidence intervals for each parental income percentile were estimated separately in the adopted children and presented in Figure 3. In the following, strengths and weaknesses with the adoption design applied in this study are discussed. This is followed by a description of international adoption in Norway, including the process of assigning adoptees to families. Finally, the design, main results, and the interpretations related to this part of the analysis are discussed.

Several explanations for the associations between parental income and children’s mental disorders have been proposed.^3^ Simplified, the social causation hypothesis implies that low income causes mental disorders, for instance through stress or poor material conditions, whereas the social selection hypothesis suggests that shared characteristics, such as genetic predispositions or area of living, influence both mental disorders in children and the parents’ income.^4^ The selection theory is supported from research suggesting there are genetic components in both income and mental disorders, and a correlation between these.^5^ Studies suggest that there is a genetic component in the intergenerational transmission of mental disorders ^6-8^ ^9^ and in the transmission from parental educational attainment to child psychopathology.^10^ The research to date has been limited in its ability to distinguish between selection and causation in the intergenerational correlation of wealth (for a recent review, see Reiss ^3^). However, sorting out these scenarios and their relative importance to mental disorders is central to understand how economic conditions or government policies may reduce income differentials in mental disorders.

Adoption designs, which is an approach frequently applied in behavioral genetics, have been used to study and break down the contribution of genetics, family environment and non-shared environment to behaviors and disorders.^11-14^ The quasi-random assignment of adoptees to adoptive families provides a unique opportunity to study associations between different family environments and children’s mental disorders. The aim of the current study is to use adoption data to estimate parts of the associations between family environment and specific outcome variables, in the absence of a genetic connection between parents and children.^13^ We do not aim at biometric variance decomposition.

The specificity of the adoption setting raises questions about whether the associations are unique to adoptive parents and their adopted children, or if they are possible to generalize to a larger population of parents and children. One difficulty with the adoption design is that, for adoptees, the assignment process is not completely random^12^. Without random assignment, the relation between the parental income and mental disorders of the children may no longer be independent of inherited abilities and traits.^15 16^ Non-random matches involve both related (i.e. adoptions within the extended family) and unrelated adoptions (for example international adoption). In case of the unrelated adoptions used in the current study, non-random matches occur less frequent.^13 15^ However, it are still possible if high income parents seek to adopt children with more fortunate backgrounds or if adoption agencies use corresponding qualities of both natural and adoptive parents as a matching strategy.^17 18^ Therefore, some context and background information on the Norwegian adoption process and the selection of adoptive parents is provided. It seems that the adoption approach in Norway does not constitute the perfect experiment for the effect of changing the family background on that of the adopted child’s mental disorders. Nevertheless, the estimation of associations between parental income and mental disorders in a sample of adoptees is valuable. Compared to conventional estimates, this approach may reduce the bias from inherited abilities and traits.^15^ Furthermore, a complementary approach that describes the same associations of parental income and mental disorders in the absence of inherited ability and assortative mating, may increase the understanding of the associations.

*International adoption in Norway*

The three countries with the largest share of inter-country adoptions to Norway from 1980 until 2016 were South Korea, Columbia and China.^19^ More boys than girls have been adopted from all the major adoption countries. Traditionally, it was mainly girls that were adopted from China. However, the number of boys adopted from China has increased every year since 2009, while the number of adopted girls has fallen. Eight out of ten children adopted to Norway from abroad were under 5 years old, and 44 per cent were in the age group 0-2 years. ^19^

*Adoption procedures*

Both the Norwegian government and the adoption agencies combine sociodemographic characteristics and judgement to assess parents for adoption approval. Any family (or single person) in Norway who wants to adopt a child from abroad, must apply for an advance approval, issued by the Regional Office for Children, Youth and Family Affairs, before the application can be sent to the country of origin of the child.^20^ The Regional Office for Children, Youth and Family Affairs will make a home study of the family, interview them and write a social report on the applicants, consisting of two parts: 1) a summary of the applicant’s background, marriage, everyday life, interests, motives for wanting to adopt a child from abroad etc; and, 2) a description of the social worker’s impressions of the family and their capabilities to become parents by adoption. The report must conclude on a recommendation whether the family should be given an advance approval to adopt a child or not. This recommendation is to be based on what could be considered in the best interest of an unknown child.^20^

In addition to the social report, the application must consist of the applicants’ health certificates, police records, marriage and birth certificates and documentation of their financial position.

When the application is completed, the competent authority is responsible for sending it to another department in the Regional Office for Children, Youth and Family Affairs for processing. The applicants must register themselves as members in one of the three accredited adoption associations before the application will be decided upon. The adoption associations are responsible for arranging adoptions to the applying families in Norway, if the application for advance approval is granted.

*Methods for the adoption study*

The study of mental disorders by parental income in the adopted children was approved and participant consent was waived by the Regional Committee for Medical and Health Research Ethics South-East Norway, reference number 2013/2394.

Data sources and study population

Linked individual-level information from five Norwegian national registries provided the basis for identification of the study population: the Population Register, the National Registry for Personal Taxpayers, the National Education Database, primary health care data from the Norwegian Control and Distribution of Health Reimbursement (KUHR) database, and specialist health care data from the National Patient Registry (NPR).

Children were assumed to be international adoptees if they were born in South-Korea, China or Columbia^19^ and fulfilled two additional criteria: only children who had parents and grandparents that were all Norwegian born were classified as adopted; and, their Norwegian born (adoptive) parents had to be registered as living in Norway the year the child was born.

Statistical methods

One-year prevalence of mental disorders with 95% confidence intervals for each parental income percentile were estimated separately for the adopted children. These estimations were based on the same model as used for the Norwegian born children, which meant pooling data across all years, using generalized estimating equations (GEE) with a logit link function and an independent working correlation structure. Robust standard errors were used to account for multiple observations of the same individual across years.^1^

The models used to estimate associations included a set of child specific control variables to account for cohort and age effects by source country. These were the following: age, sex and birth year interacted with country of birth. In addition, the age of the child the year it was registered with a Norwegian citizenship was adjusted for. In a sensitivity analysis, only children who were registered as Norwegian citizens prior to the age of two were included.

The first model, where parental income percentile is included as a continuous variable can be written as:

$logit\{Pr \left( {MentalDisorder}_{it}=1 | x_{it} \right)\}=\alpha+\beta_{1}{IncDecile}_{it}+X_{it}^{'}\gamma$,

The second model, where parental income decile is included as included as 9 dummy variables (omitting category 1) can be written as:

$logit\{Pr \left( {MentalDisorder}_{it}=1 | x_{it} \right)\}=\alpha+\beta_{1}{IncDecile2}_{it}+\ldots+\beta_{9}{IncDecile10}_{it}+X_{it}^{'}\gamma$, (5)

where ${MentalDisorder}_{it}$is a dummy variable that is equal to 1 when the child had any mental disorder and zero otherwise in year. $X_{it}^{'}$ is a vector of control variables for: age of Norwegian citizenship; age; sex; and birth year interacted with country of birth.

*Results*

With the exception of eating disorders, international adoptees had around 1.5-2.0 times higher prevalence of all diagnosed mental disorders, compared with Norwegian born children (Figure 3, Table S1). The associations between parental income and children’s mental disorders were negative for total mental disorders, ADHD, anxiety, and depression. The associations between parental income and children’s mental disorders were more pronounced in Norwegian born children, compared with international adoptees. For total mental disorders, the slope of the association between parental income and mental disorders of -2.54 (95%CI, -4.65 -0.43), was less steep than the slope in the Norwegian born children of -6.56 (95%CI, -6.67 -6.45).

*Interpretation*

The study findings demonstrate that adoptees raised in low-income families had a higher prevalence of mental disorders, compared to adoptees raised in high-income families. This suggests that the associations of parental income and children’s mental disorders were not merely due to selection. However, associations between parental income and mental disorders were less pronounced than those seen among children living with biological parents.

Care is required when comparing the associations of parental income and mental disorders in the adoptees with the Norwegian born population as the adopted had more mental disorders, in accordance with prior literature.^21 22^ In addition, the selection of adoptive parents based on sociodemographic characteristics and interviews excludes the most deprived families from the adoption sample.

**Part III: Variation in the association between mental distress and health service use by parental income in children and adolescents**

Health registry data are limited to individuals who have been in contact with the health services and many individuals with mental disorders will go untreated. If the proportion of untreated children varies by parental income, the findings of the current study will be biased. Hence, the aim of the following analysis was to explore the association between mental distress and health service use, by parental income.

Data & Variables

This analysis used the Survey of Health and Living Conditions (SHLC), a cross-sectional health survey, with data collected in 2005 by Statistics Norway.^23^ The survey is a nationally representative health survey of individuals in Norway over the age of 15. Statistics Norway draws a random sample of 10 000 individuals each year and then excludes individuals who have died or have moved abroad. In addition, individuals living in the following institutions are excluded from the data collection: retirement homes, combined hospitals and retirement homes, orphanages, youth homes, psychiatric hospitals and nursing homes, institutions for those with developmental disabilities, and institutions for individuals with alcohol- and drug use disorders. The exclusion of these individuals sum up to around 200 individuals, thus individuals with severe mental and somatic health problems who are not able to live independently, and the oldest age-groups are under-represented in this survey.

The survey includes questions about all household members. In 2005, all individuals with children aged 6-15 were asked additional questions about their children mental distress and health service use.

Psychological distress was measured by an 8 item version of the Hopkins Symptom Check List (HSCL-8), which is a mental health screening instrument primarily measuring symptoms of anxiety and depression.^24^ The 8 items were selected from the 25 item version of the HSCL. Mothers and fathers are also asked to describe whether or not their children has had a number of symptoms from the HSCL-8 and how often, during the past 3 months. The mental distress symptoms were: 1) nervousness or shakiness inside; 2) difficulties falling asleep; 3) feeling blue; 4) feeling lonely; 5) headaches; 6) feeling restless, can’t sit still; 7) feeling tense or keyed up; and, 8) suddenly scared for no reason. The parents were asked to indicate the regularity of each symptom on the scale: a) every day, b) more than once a week, c) once a week, d) once a month, e) less or never.

Based on the responses of these symptoms questions, two variables were generated. First, a score from zero to 32, where each a-response was coded with the value 4, b-response 3, c-response 2, d-response 1 and e-response 0. Second, a dummy variable was generated, coded one if a or b was the response to any of the symptoms, and zero otherwise.

Health service use was coded as 1 if the child had visited any general practitioner, specialist physician outside of hospital, specialist physician at hospital, psychologist or mental health facilities for children and adolescents during the last year, and zero otherwise.

Household income was merged with the survey data from the National Registry for Personal Taxpayers.

The participants gave their informed consent at the time of the telephone interview.

Statistical analysis

To account for missing responses in the sample, weights provided in the survey were used. These were calculated based on gender, age, education, and family size.^23^

Odds ratios were estimated based on a logistic regression model of the binary dependent variable for health service use on the mental distress score (and dummy mental distress problems) and household income, adjusting for age and sex. The model was run again including interactions between income and mental distress. All regressions were also run using linear probability models.

Result

Children and adolescents with a higher psychological distress score had a higher log odds for utilizing health services during one year (Table S2). No significant effect was detected between income and health service use. Furthermore, no significant interaction was found between the mental distress score and health service use. Similar results were found when treating the mental distress score as a dummy (Table S3).

Interpretation

These findings suggest that with the same level of psychological distress, there was no difference in the propensity to use health services for children and adolescents by parental income, in 2005.

**Part IV: The proportion of variance in the child-adolescent mental disorders predictable by income and education**

To explore the proportion variance in child-adolescent mental disorders explained by parental education relative to parental income, the proportion with a mental disorder by seven parental education categories was estimated (Figure S5). The education categories are based on the parent with the highest education (Table S4). Second, 7 groups by parental income was generated to match the percentages of individuals within each education group. For instance, when 4.6 % of children-adolescents were in the lowest education group, 4.6% of children were also placed in the lowest income group (Table S4).

To estimate the proportion of variance in the child-adolescent mental disorders predictable by income and education, two linear probability models were fitted. One regresses mental disorder (yes/no) on the 7 parental education categories, adjusted for age and sex. The second regresses mental disorder (yes/no) on the 7 parental income categories, adjusted for age and sex. The R^2^ from the “education model” was 0.0195. The R^2^ from the “income model” was 0.209.

Interpretation: Parental income explains slightly more of the variance in child-adolescent mental disorders than parental education.

**Part V: Comparison of Diagnosed Mental Disorder by Immigrant Categories**

Comparisons of the diagnostic overlap between the NPR and KUHR with the Composite International Diagnostic Interview (CIDI) for the assessment of the DSM‐IV and ICD‐10 in Norway, estimated that the registries have moderate sensitivity as indicators of the interview based diagnoses, but excellent specificity with between 0.2% and 4.2% false positives for anxiety and depression.^25 26^ Part 1 above suggests that the sensitivity does not vary by income. However, there are reasons to suspect systematic variations in sensitivity for some immigrant groups.

In Norway, immigrant women are found to be less likely to consult with their general practitioner for mental disorders than non-immigrant women, but refugees have more mental health contacts in primary healthcare services than non-refugee immigrants from the same country.^26 27^ Similarly, other studies have found that children and adolescents from Eastern parts of Europe (except Russia) and from non-Western backgrounds (except for Iran) tend to underutilize specialist mental healthcare in Norway.^28^ Factors associated with the use of services among immigrants are: years since migration, the health care system in the source country and the gross domestic product in the source country. It has been argued that poor knowledge of the health care system, inability to make appointments by phone (language barriers) and lack of a regular general practitioner may contribute to this.^29^

Immigrants also has lower wages, compared with non-immigrants.^30^ Hence, the inclusion of immigrants in the study population would bias the estimated associations between parental income and mental disorders as this would have included a population with lower wages and lower sensitivity.

Mental health care use in four groups classified by immigrant status according to Statistics Norway Country group and immigrant classification was explored.^31 32^

Group 1: Norwegian born with parents who immigrated from EU/EEA, USA, Canada, Australia and New Zealand.

Group 2: Born in EU/EEA, USA, Canada, Australia and New Zealand.

Group 3: Norwegian born with parents who immigrated from Asia, Africa, Latin America, Oceania except Australia and New Zealand and Europa excluding EU/EEA.

Group 4: Born in Asia, Africa, Latin America, Oceania except Australia and New Zealand and Europa excluding EU/EEA.

Table S5 demonstrates differences in the prevalence of mental disorders by immigrant background. For example, the share of the population who was registered with Psychotic disorders (including Schizophrenia) was more than twice as high in the children born in Asia, Africa, Latin America, Oceania except Australia and New Zealand and Europa excluding EU/EEA, compared with the children born in Norway. However, depression was lower for this group, compared with children born in Norway. Figure S3 shows the association between parental income and mental disorders for immigrants. No clear pattern emerges. The group includes all immigrants with varying years since migration and source countries, which should be kept in mind when considering this result.

**Part VI: International Classification of Primary Care, Second edition (ICPC-2)**

The ICPC classification was first developed in 1987 by WONCA International Classification Committee, then as HICPIC. The ICPC-2 version, which is the second revision and used in this study, was last updated in March 2003.^33^ The World Health Organization (WHO) has accepted ICPC-2 within the WHO Family of International Classifications. It is used as a classification for primary care or general practice wherever applicable.

ICPC-2 classifies patient data and clinical activity in primary care and allows classification of the patient’s reason for encounter, the problems/diagnosis managed, interventions, and the ordering of these data in an episode of care structure.^33^

*Classification Structure*

The ICPC-2 contains 17 chapters: A General and unspecified; B Blood, blood forming organs, lymphatics, spleen; D Digestive; F Eye; H Ear; K Circulatory; L Musculoskeletal; N Neurological; P Psychological; R Respiratory; S Skin; T Endocrine, metabolic and nutritional; U Urology; W Pregnancy, childbirth, family planning; X Female genital system and breast; Y Male genital system; Z Social problems. Each chapter is divided into 7 components dealing with symptoms and complaints (component 1), diagnostic, screening and preventive procedures (component 2), medication, treatment and procedures (component 3), test results (component 4), administrative (component 5), referrals and other reasons for encounter (component 6) and diseases (component 7).

**Part VII: Associations between Children’s Diagnosed Mental Disorders and Local Area Income Inequality and Poverty**

A theory is that mental disorders is related to income inequality, which may increase stress for low-income individuals.^34^

Income inequality and distribution was estimated using three area-level measures, generated by Statistics Norway. These were: the Gini-coefficient, the ratio of the upper bound value of the ninth decile to that of the first decile (P90/P10), and on the proportion below the OECD 60 poverty line. Higher numbers indicate more unequal income distributions.

Adjusted ORs for a diagnosed mental disorder by area level characteristics were estimated by regressing the one-year prevalences of mental disorders on the Gini-coefficient, P90/P10, and on the proportion below the OECD 60 poverty line, within 133 regions of residence defined by city-districts and municipalities in Norway, using multilevel mixed-effects logistic regression and area level fixed effects logit regression models. Regions with less than 1,000 inhabitants were excluded from this analyses. Two versions of each multilevel model was estimated: first, adjusted for calendar year, age, age squared, sex and interactions between age and sex; and, second, adjusted for parental income and aggregate area income.

Increased Gini-coefficient was significantly associated with reduced mental disorders in children (Figure 6). However, in the models adjusting for parental income and aggregate area income, no association was found. Mental disorder in children was not significantly associated with P90/P10 or OECD60 poverty in any of the models (Figure 6).

*Interpretation*

There was no association between inequality and mental disorders across areas.

**References**

1. Liang K-Y, Zeger SL. Longitudinal data analysis using generalized linear models. *Biometrika* 1986;73(1):13-22.

2. Cameron AC, Miller DL. A practitioner’s guide to cluster-robust inference. *Journal of human resources* 2015;50(2):317-72.

3. Reiss F. Socioeconomic inequalities and mental health problems in children and adolescents: a systematic review. *Social science & medicine* 2013;90:24-31.

4. Murali V, Oyebode F. Poverty, social inequality and mental health. *Advances in Psychiatric Treatment* 2004;10(3):216-24. doi: 10.1192/apt.10.3.216

5. Hill WD, Hagenaars SP, Marioni RE, et al. Molecular genetic contributions to social deprivation and household income in UK Biobank. 2016;26(22):3083-89.

6. Heston LL. Psychiatric disorders in foster home reared children of schizophrenic mothers. *The British journal of psychiatry : the journal of mental science* 1966;112(489):819-25. doi: 10.1192/bjp.112.489.819 [published Online First: 1966/08/01]

7. Goodwin DW, Schulsinger F, Hermansen L, et al. Alcohol Problems in Adoptees Raised Apart From Alcoholic Biological Parents. *Archives of General Psychiatry* 1973;28(2):238-43. doi: 10.1001/archpsyc.1973.01750320068011 %J Archives of General Psychiatry

8. Cadoret RJ, Yates WR, Ed T, et al. Genetic-Environmental Interaction in the Genesis of Aggressivity and Conduct Disorders. *Archives of General Psychiatry* 1995;52(11):916-24. doi: 10.1001/archpsyc.1995.03950230030006 %J Archives of General Psychiatry

9. Kendler KS, Ohlsson H, Sundquist K, et al. Sources of Parent-Offspring Resemblance for Major Depression in a National Swedish Extended Adoption Study. *JAMA Psychiatry* 2018;75(2):194-200. doi: 10.1001/jamapsychiatry.2017.3828 %J JAMA Psychiatry

10. Torvik FA, Eilertsen EM, McAdams TA, et al. Mechanisms linking parental educational attainment with child ADHD, depression, and academic problems: a study of extended families in The Norwegian Mother, Father and Child Cohort Study.n/a(n/a) doi: 10.1111/jcpp.13197

11. Boag PT, van Noordwijk AJJAgAP, London. Quantitative genetics. 1987:45-78.

12. Spinath FM, Johnson W. Behavior genetics. 2011

13. Sacerdote B. Nature and Nurture Effects on Children’s Outcomes: What Have We Learned from Studies of Twins and Adoptees? Handbook of social economics: Elsevier 2011:1-30.

14. Biron P, Mongeau JG, Bertrand D. Familial aggregation of blood pressure in 558 adopted children. *Can Med Assoc J* 1976;115(8):773-74.

15. Plug EJAER. Estimating the effect of mother's schooling on children's schooling using a sample of adoptees. 2004;94(1):358-68.

16. Plug E, Vijverberg W. Does family income matter for schooling outcomes? Using adoptees as a natural experiment. *The Economic Journal* 2005;115(506):879-906.

17. Modell JS. Kinship with strangers: Adoption and interpretations of kinship in American culture: University of California Press Berkeley 1994.

18. Melosh B. Strangers and kin: Harvard University Press 2002.

19. Statistics Norway. Adoptions: Statistics Norway, 2019.

20. InorAdopt. The adoption procedureInorAdopt: InorAdoptInorAdopt; [Available from: <https://inoradopt.no/adoption-procedure/> accessed Jan 22 2020.

21. Hjern A, Lindblad F, Vinnerljung B. Suicide, psychiatric illness, and social maladjustment in intercountry adoptees in Sweden: a cohort study. *The lancet* 2002;360(9331):443-48.

22. Juffer F, Van Ijzendoorn MH. Behavior problems and mental health referrals of international adoptees: A meta-analysis. *Jama* 2005;293(20):2501-15.

23. Hougen HC. Survey of Health and Living Conditions [in Norwegian]. In: Norway S, ed. Oslo: Statistics Norway, 2006.

24. Derogatis LR, Lipman RS, Rickels K, et al. The Hopkins Symptom Checklist (HSCL): a self-report symptom inventory. *Behavioral science* 1974;19(1):1-15. [published Online First: 1974/01/01]

25. Torvik FA, Ystrom E, Gustavson K, et al. Diagnostic and genetic overlap of three common mental disorders in structured interviews and health registries. *Acta Psychiatrica Scandinavica* 2018;137(1):54-64.

26. Straiton ML, Reneflot A, Diaz E. Mental Health of Refugees and Non-refugees from War-Conflict Countries: Data from Primary Healthcare Services and the Norwegian Prescription Database. *Journal of Immigrant and Minority Health* 2017;19(3):582-89. doi: 10.1007/s10903-016-0450-y

27. Straiton M, Reneflot A, Diaz E. Immigrants’ use of primary health care services for mental health problems. *BMC Health Services Research* 2014;14(1):341. doi: 10.1186/1472-6963-14-341

28. Abebe DS, Lien L, Elstad JI. Immigrants’ utilization of specialist mental healthcare according to age, country of origin, and migration history: a nation-wide register study in Norway. *Social Psychiatry and Psychiatric Epidemiology* 2017;52(6):679-87. doi: 10.1007/s00127-017-1381-1

29. Sandvik H, Hunskaar S, Diaz E. Immigrants’ use of emergency primary health care in Norway: a registry-based observational study. *BMC Health Services Research* 2012;12(1):308. doi: 10.1186/1472-6963-12-308

30. Statistics Norway. Lower wages among immigrants [in Norwegian] Oslo, Norway: Statistics Norway; 2017 [Available from: <https://www.ssb.no/arbeid-og-lonn/artikler-og-publikasjoner/lavere-lonn-blant-innvandrere>.

31. Statistics Norway. Classification of immigration categories: Statistics Norway; 2008 [Available from: <https://www.ssb.no/en/klass/klassifikasjoner/82>.

32. Statistics Norway. Country and citizenship in population statistics 2011-12: Statistics Norway; 2011 [Available from: <https://www.ssb.no/en/klass/klassifikasjoner/91/varianter/847>.

33. Published. WHOJ. International Classification of Primary Care (ICPC-2) Disponible en: <http://www>. who. int/classifications/icd/adaptations/icpc2/en. 2016

34. Pickett KE, Wilkinson RG. Inequality: an underacknowledged source of mental illness and distress. *The British Journal of Psychiatry* 2010;197(6):426-28.

**Table S1**.Sample characteristics

|  | **Main estimation sample** | | |  | **Immigrant background sample** | | |  | **International adoptees sample** | | |
| --- | --- | --- | --- | --- | --- | --- | --- | --- | --- | --- | --- |
| Number of individuals | 969206 |  |  |  | 293647 |  |  |  | 5698 |  |  |
| Person years | 5199742 |  |  |  | 1461554 |  |  |  | 36281 |  |  |
|  |  |  | |  |  |  |  |  |  |  |  |
| *Child mental disorders* | *Mean* | *(95 % CI)* | |  | *Mean* | *(95 % CI)* |  |  | *Mean* | *(95 % CI)* |  |
| Total mental disorders | 6.46 % | (6.44 6.48) | |  | 5.27 % | (5.23 5.30) | |  | 10.13 % | (9.83 10.43) | |
| Substance use | 0.07 % | (0.07 0.07) | |  | 0.06 % | (0.05 0.06) | |  | 0.11 % | (0.07 0.14) | |
| Psychotic disorders (including Schizophrenia) | 0.05 % | (0.05 0.05) | |  | 0.06 % | (0.06 0.07) | |  | 0.08 % | (0.05 0.11) | |
| Bipolar disorder | 0.03 % | (0.03 0.03) | |  | 0.03 % | (0.02 0.03) | |  | 0.06 % | (0.04 0.08) | |
| Depressive disorders | 0.79 % | (0.78 0.80) | |  | 0.59 % | 0.58 0.61) | |  | 1.04 % | (0.94 1.14) | |
| Anxiety disorders | 1.26 % | (1.25 1.27) | |  | 1.11 % | (1.10 1.13) | |  | 1.58 % | (1.46 1.70) | |
| Somatoform disorders | 0.08 % | (0.08 0.08) | |  | 0.08 % | (0.07 0.08) | |  | 0.11 % | (0.08 0.15) | |
| Eating disorders | 0.14 % | (0.14 0.14) | |  | 0.09 % | (0.09 0.10) | |  | 0.10 % | (0.07 0.13) | |
| Idiopathic developmental intellectual disability | 0.30 % | (0.30 0.31) | |  | 0.42 % | (0.41 0.43) | |  | 0.55 % | (0.47 0.62) | |
| Autism spectrum disorders | 0.45 % | (0.44 0.45) | |  | 0.47 % | (0.46 0.48) | |  | 0.63 % | (0.55 0.71) | |
| Attention-deficit/hyperactivity disorder | 2.72 % | (2.70 2.73) | |  | 1.62 % | (1.60 1.64) | |  | 4.29 % | (4.09 4.49) | |
| Conduct disorder | 0.27 % | (0.27 0.28) | |  | 0.26 % | (0.25 0.27) | |  | 0.45 % | (0.39 0.52) | |
| Other with onset in childhood | 0.97 % | (0.97 0.98) | |  | 0.85 % | (0.83 0.86) | |  | 2.16 % | (2.02 2.30) | |
| Mental disorder, not otherwise specified | 0.61 % | (0.60 0.62) | |  | 0.65 % | (0.63 0.66) | |  | 0.88 % | (0.78 0.97) | |
| Other mental disorders | 0.43 % | (0.43 0.44) | |  | 0.43 % | (0.42 0.44) | |  | 0.78 % | (0.69 0.86) | |
|  |  |  |  |  |  |  |  |  |  |  |  |
| *Child age and sex* | *Mean* | *(95 % CI)* |  |  | *Mean* | *(95 % CI)* |  |  | *Mean* | *(95 % CI)* |  |
| Age (mean) | 11.17 | (11.17 11.17) | |  | 10.68 | (10.68 10.69) | |  | 11.14 | (11.10 11.17) | |
| Percent female | 48.70 % | (48.66 48.74) | |  | 49.01 % | (48.93 49.08) | |  | 65.59 % | (65.12 66.05) | |
|  |  |  |  |  |  |  |  |  |  |  |  |
| *Parental characteristics* | *Mean* | *(95 % CI)* |  |  | *Mean* | *(95 % CI)* |  |  | *Mean* | *(95 % CI)* |  |
| Parent diagnosed with mental disorders | 18.00 % | (17.97 18.03) | |  | 18.25 % | (18.19 18.31) | |  | 13.38 % | (13.05 13.72) | |
| Father age | 43.44407 | (43.44 3.45) | |  | 44.44268 |  | |  | 49.6537 | (49.60 49.71) | |
| Mother age | 40.76216 | (40.76 0.77) | |  | 40.16272 | (40.15 40.17) | |  | 47.97637 | (47.92 48.03) | |
| At least one parent with college or university degree | 55.75 % | (55.71 5.79) | |  | 48.7 | (48.63 48.78) | |  | 69.87 | (69.42 70.32) | |
| Parental income (mother + fathers income) in 2015 NOK | 805016 | (804266 805765) | |  | 689464 | (687703 691225) | |  | 873559 | (864652 882467) | |
| Parents in the same household | 72.46 % | (72.43 72.50) | |  | 70.39 % | (70.32 70.45) | |  | 85.97 % | (85.63 86.31) | |

**Table S2.** Adjusted Odds Ratios for Health Service Use for Participants by Mental Distress Score, Parental Income and Interactions between Mental Distress Scores and Parental Income^a^

|  | Model 1 |  | Model 2 |  | Model 3 |  | Model 4 |
| --- | --- | --- | --- | --- | --- | --- | --- |
| Mental distress score | 1.11 [1.08 1.15] |  |  |  | 1.11 [1.08 1.15] |  | 1.12 [1.05 1.19] |
| Household income decile |  |  | 0.98 [0.95 1.02] |  | 1.00 [0.96 1.04] |  | 1.00 [0.95 1.05] |
| ScoreXincome |  |  |  |  |  |  | 1.00 [0.99 1.05] |
|  |  |  |  |  |  |  |  |
| *Mean health service use [95% CI]* | *0.53 [0.50 0.55]* | | | | | | |
| *N* | *1,431* | | | | | | |

^a^ The odds ratios were estimated using logistic regression models adjusted for age and sex.

**Table S3**. Adjusted Odds Ratios for Health Service Use for Participants by Mental Distress Dummy, Parental Income and Interactions between Mental Distress Dummy and Parental Income^a^

|  | Model 1 |  | Model 2 |  | Model 3 |  | Model 4 |
| --- | --- | --- | --- | --- | --- | --- | --- |
| Mental Distress dummy | 1.91 [1.51 2.43] |  |  |  | 1.90 [1.50 2.42] |  | 1.81 [1.10 2.98] |
| Household income decile | |  | 0.98 [0.95 1.02] | | 0.99 [0.96 1.03] |  | 0.99 [0.95 1.03] |
| DummyXincome | |  |  |  |  |  | 1.01 [0.93 1.10] |
|  |  |  |  |  |  |  |  |
| *Mean health service use [95% CI]* | *0.53 [0.50 0.55]* | | | | | | |
| *N* | *1,431* | | | | | | |

^a^ The odds ratios were estimated using logistic regression models adjusted for age and sex.

**Table S4.** Percent of the main estimation sample within each education and income group

| **Parental education** | **Percent within each category** |
| --- | --- |
| Lower secondary | 4.62 |
| Upper secondary, basic | 3.03 |
| Upper secondary, final year | 30.52 |
| Post-secondary non-tertiary | 5.08 |
| Undergrad level | 40.73 |
| Grad level | 14.32 |
| Postgraduate (PhD) | 1.7 |
|  |  |
| **Parental income categories** | **Percent within each category** |
| Income category 1 | 4.61 |
| Income category 2 | 3 |
| Income category 3 | 30.53 |
| Income category 4 | 5.11 |
| Income category 5 | 40.64 |
| Income category 6 | 14.41 |
| Income category 7 | 1.7 |

**Table S5.** Mental disorders by immigrant background

|  | Main estimation sample | | |  | Norwegian born with parents who immigrated from EU/EEA, USA, Canada, Australia and New Zealand | | |  | Born in EU/EEA, USA, Canada, Australia and New Zealand. | | |  | Norwegian born with parents who immigrated from Asia, Africa, Latin America, Oceania except Australia and New Zealand and Europa excluding EU/EEA. | | |  | Born in Asia, Africa, Latin America, Oceania except Australia and New Zealand and Europa excluding EU/EEA. | | |
| --- | --- | --- | --- | --- | --- | --- | --- | --- | --- | --- | --- | --- | --- | --- | --- | --- | --- | --- | --- |
| Male | Mean | (95% CI) | |  | Mean | (95% CI) | |  | Mean | (95% CI) | |  | Mean | (95% CI) | |  | Mean | (95% CI) | |
| Total mental disorders | 7.435 | (7.374 7.496) | |  | 6.892 | (6.686 7.099) | |  | 6.051 | (5.804 6.297) | |  | 5.668 | (5.520 5.817) | |  | 6.728 | (6.496 6.959) | |
| Substance use | 0.064 | (0.061 0.068) | |  | 0.059 | (0.048 0.069) | |  | 0.054 | (0.041 0.067) | |  | 0.043 | (0.036 0.050) | |  | 0.097 | (0.080 0.113) | |
| Psychotic disorders (including Schizophrenia) | 0.051 | (0.047 0.055) | |  | 0.055 | (0.037 0.072) | |  | 0.049 | (0.027 0.071) | |  | 0.069 | (0.054 0.083) | |  | 0.109 | (0.086 0.132) | |
| Bipolar disorder | 0.030 | (0.027 0.034) | |  | 0.038 | (0.023 0.053) | |  | 0.023 | (0.006 0.040) | |  | 0.014 | (0.007 0.021) | |  | 0.033 | (0.020 0.047) | |
| Depressive disorders | 0.479 | (0.468 0.490) | |  | 0.472 | (0.433 0.511) | |  | 0.435 | (0.388 0.483) | |  | 0.286 | (0.261 0.310) | |  | 0.466 | (0.424 0.507) | |
| Anxiety disorders | 0.997 | (0.979 1.015) | |  | 1.007 | (0.942 1.071) | |  | 0.814 | (0.739 0.888) | |  | 0.811 | (0.764 0.858) | |  | 1.434 | (1.346 1.522) | |
| Somatoform disorders | 0.057 | (0.054 0.060) | |  | 0.066 | (0.054 0.079) | |  | 0.045 | (0.032 0.057) | |  | 0.054 | (0.045 0.063) | |  | 0.065 | (0.050 0.080) | |
| Eating disorders | 0.033 | (0.030 0.036) | |  | 0.031 | (0.020 0.042) | |  | 0.029 | (0.017 0.041) | |  | 0.026 | (0.019 0.033) | |  | 0.034 | (0.021 0.047) | |
| Idiopathic developmental intellectual disability | 0.335 | (0.321 0.348) | |  | 0.325 | (0.278 0.373) | |  | 0.360 | (0.297 0.422) | |  | 0.627 | (0.576 0.678) | |  | 0.711 | (0.633 0.788) | |
| Autism spectrum disorders | 0.677 | (0.658 0.695) | |  | 0.793 | (0.723 0.863) | |  | 0.699 | (0.615 0.784) | |  | 0.807 | (0.748 0.866) | |  | 0.581 | (0.507 0.655) | |
| Attention-deficit/hyperactivity disorder | 3.815 | (3.766 3.863) | |  | 3.008 | (2.856 3.160) | |  | 2.656 | (2.477 2.835) | |  | 1.834 | (1.741 1.927) | |  | 2.315 | (2.155 2.474) | |
| Conduct disorder | 0.371 | (0.359 0.383) | |  | 0.410 | (0.364 0.456) | |  | 0.323 | (0.270 0.376) | |  | 0.344 | (0.312 0.375) | |  | 0.379 | (0.330 0.427) | |
| Other with onset in childhood | 1.275 | (1.253 1.297) | |  | 1.215 | (1.138 1.292) | |  | 1.025 | (0.932 1.118) | |  | 0.895 | (0.845 0.945) | |  | 1.278 | (1.183 1.374) | |
| Mental disorder, not otherwise specified | 0.731 | (0.714 0.749) | |  | 0.812 | (0.745 0.879) | |  | 0.766 | (0.684 0.848) | |  | 0.957 | (0.894 1.021) | |  | 0.770 | (0.695 0.845) | |
| Other mental disorders | 0.445 | (0.433 0.456) | |  | 0.454 | (0.414 0.493) | |  | 0.445 | (0.391 0.500) | |  | 0.503 | (0.470 0.537) | |  | 0.537 | (0.487 0.586) | |
|  |  |  |  |  |  |  |  |  |  |  |  |  |  |  |  |  |  |  |  |
| Female |  |  |  |  |  |  |  |  |  |  |  |  |  |  |  |  |  |  |  |
| Total mental disorders | 5.440 | (5.392 5.488) | |  | 4.771 | (4.610 4.933) | |  | 4.155 | (3.963 4.348) | |  | 3.609 | (3.499 3.719) | |  | 4.948 | (4.765 5.131) | |
| Substance use | 0.070 | (0.067 0.074) | |  | 0.049 | (0.039 0.060) | |  | 0.065 | (0.049 0.080) | |  | 0.043 | (0.035 0.052) | |  | 0.071 | (0.057 0.085) | |
| Psychotic disorders (including Schizophrenia) | 0.049 | (0.045 0.053) | |  | 0.057 | (0.040 0.074) | |  | 0.051 | (0.032 0.071) | |  | 0.045 | (0.035 0.054) | |  | 0.079 | (0.060 0.098) | |
| Bipolar disorder | 0.036 | (0.033 0.040) | |  | 0.033 | (0.023 0.044) | |  | 0.043 | (0.025 0.061) | |  | 0.014 | (0.008 0.020) | |  | 0.024 | (0.013 0.035) | |
| Depressive disorders | 1.120 | (1.103 1.138) | |  | 0.958 | (0.900 1.017) | |  | 0.854 | (0.784 0.924) | |  | 0.658 | (0.619 0.696) | |  | 0.878 | (0.817 0.938) | |
| Anxiety disorders | 1.530 | (1.508 1.552) | |  | 1.390 | (1.314 1.466) | |  | 1.168 | (1.082 1.255) | |  | 1.021 | (0.969 1.073) | |  | 1.575 | (1.486 1.665) | |
| Somatoform disorders | 0.109 | (0.105 0.114) | |  | 0.096 | (0.081 0.112) | |  | 0.080 | (0.060 0.099) | |  | 0.080 | (0.069 0.091) | |  | 0.133 | (0.113 0.154) | |
| Eating disorders | 0.253 | (0.244 0.262) | |  | 0.257 | (0.224 0.289) | |  | 0.181 | (0.147 0.216) | |  | 0.114 | (0.099 0.130) | |  | 0.131 | (0.109 0.154) | |
| Idiopathic developmental intellectual disability | 0.270 | (0.258 0.283) | |  | 0.150 | (0.117 0.183) | |  | 0.268 | (0.212 0.325) | |  | 0.383 | (0.341 0.424) | |  | 0.442 | (0.381 0.504) | |
| Autism spectrum disorders | 0.207 | (0.196 0.217) | |  | 0.239 | (0.198 0.279) | |  | 0.214 | (0.165 0.262) | |  | 0.167 | (0.142 0.193) | |  | 0.172 | (0.131 0.214) | |
| Attention-deficit/hyperactivity disorder | 1.563 | (1.532 1.594) | |  | 1.155 | (1.060 1.249) | |  | 1.032 | (0.918 1.145) | |  | 0.612 | (0.560 0.665) | |  | 0.903 | (0.806 1.000) | |
| Conduct disorder | 0.169 | (0.161 0.177) | |  | 0.154 | (0.128 0.180) | |  | 0.132 | (0.102 0.163) | |  | 0.141 | (0.121 0.161) | |  | 0.204 | (0.171 0.238) | |
| Other with onset in childhood | 0.658 | (0.642 0.674) | |  | 0.665 | (0.609 0.720) | |  | 0.568 | (0.496 0.641) | |  | 0.528 | (0.488 0.568) | |  | 0.780 | (0.708 0.852) | |
| Mental disorder, not otherwise specified | 0.480 | (0.467 0.492) | |  | 0.447 | (0.401 0.492) | |  | 0.441 | (0.385 0.498) | |  | 0.379 | (0.344 0.415) | |  | 0.498 | (0.446 0.551) | |
| Other mental disorders | 0.416 | 0.405 0.426 | |  | 0.397 | 0.360 0.434 | |  | 0.282 | 0.242 0.321 | |  | 0.321 | 0.295 0.347 | |  | 0.474 | 0.429 0.520 | |

**Figure S1.** Prevalence of Child-Adolescent Mental Disorders and mothers and fathers income

**
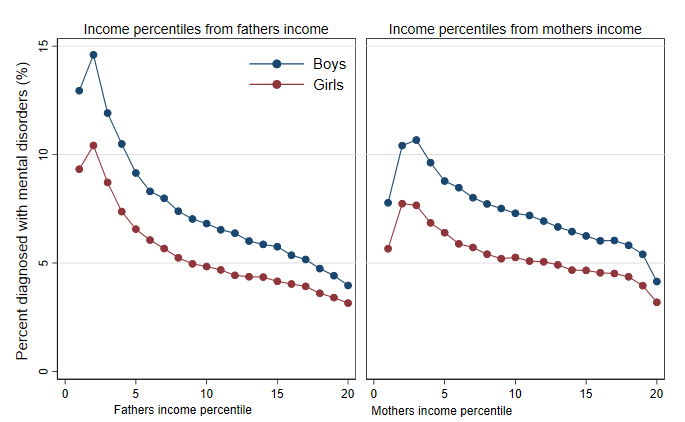
**

**Figure S2.** Prevalence of Child-Adolescent Mental Disorders and parental income percentile in the population with immigrant background

**
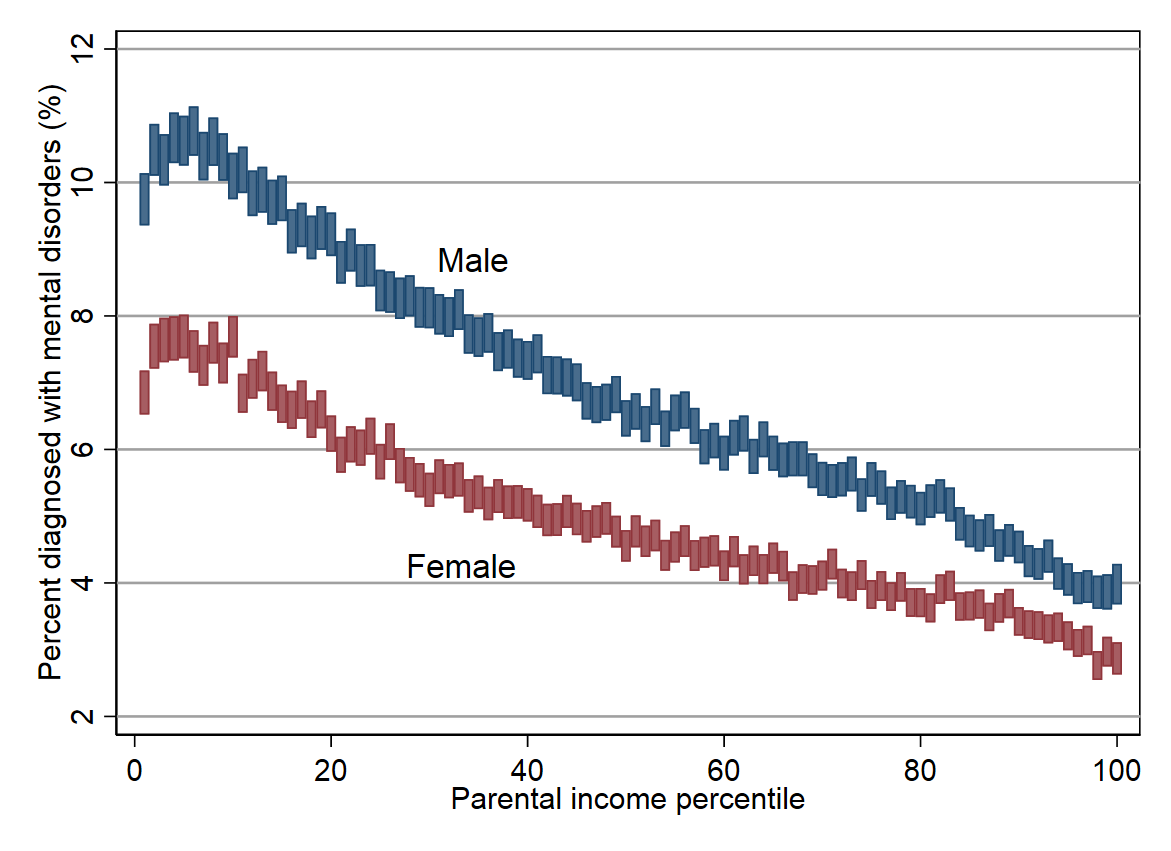
**

The vertical height of each bar represents the 95% confidence intervals

**Figure S3.** Prevalence of Child-Adolescent Mental Disorders by parental income percentile in the main estimation sample for 14 different categories of disorders.


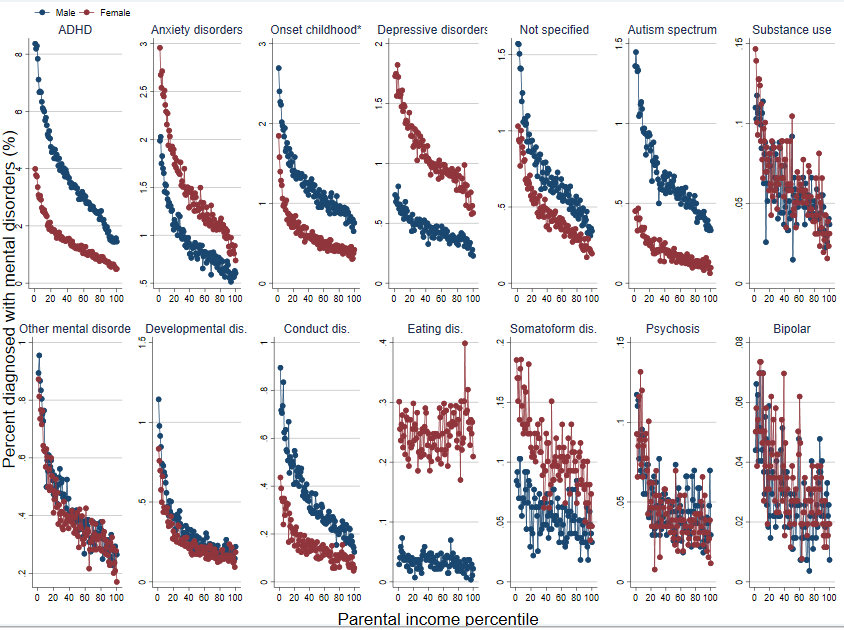


**Figure S4.** Prevalence of Child-Adolescent Mental Disorders by Parental Education*


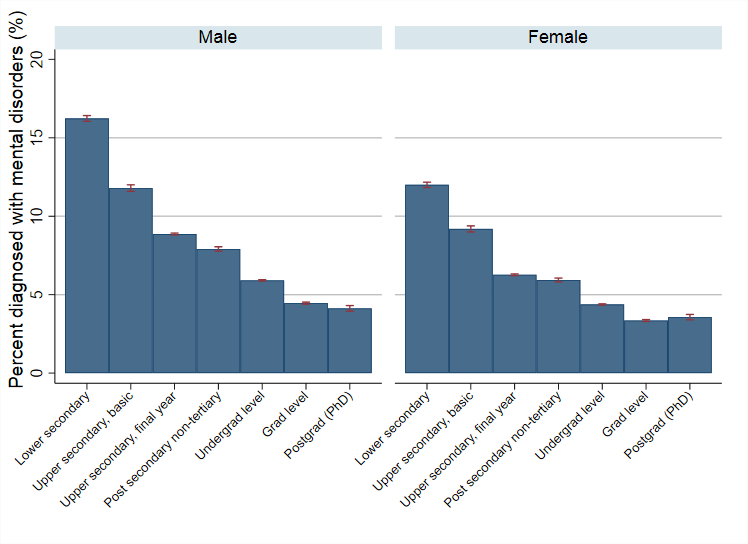


* For two-parent households, the education categories are based on the parent with the highest education

**Figure S5**. Prevalence of Child-Adolescent Mental Disorders by seven Parental Income Groups

**
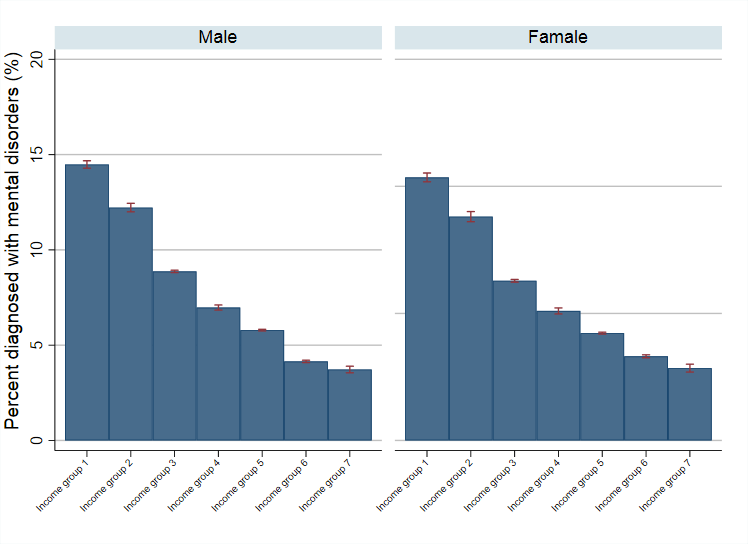
**

The proportion of individuals within each income group match the proportions within each education group (eTale 4)
